# A database of US state policies to mitigate COVID-19 and its economic consequences

**DOI:** 10.1186/s12889-022-13487-0

**Published:** 2022-06-04

**Authors:** Alexandra Skinner, Kelsey Flannery, Kristen Nocka, Jacob Bor, Lorraine T. Dean, Jonathan Jay, Sarah Ketchen Lipson, Megan B. Cole, Emily A. Benfer, Rachel Scheckman, Will Raderman, David K. Jones, Julia Raifman

**Affiliations:** 1 Boston University School of Public Health, 715 Albany Street, MB 02118 Boston, USA; 2 Johns Hopkins Bloomberg School of Public Health, Baltimore, MD USA; 3 Wake Forest School of Law, Winston-Salem, NC USA

**Keywords:** COVID-19, COVID-19 policy, Economic precarity, Mask mandate, Physical distancing, Policy, State mandate, School closure, Vaccine, Eviction moratoria, Pandemic

## Abstract

**Background:**

Since COVID-19 first appeared in the United States (US) in January 2020, US states have pursued a wide range of policies to mitigate the spread of the virus and its economic ramifications. Without unified federal guidance, states have been the front lines of the policy response.

**Main text:**

We created the COVID-19 US State Policy (*CUSP*) database (https://statepolicies.com/) to document the dates and components of economic relief and public health measures issued at the state level in response to the COVID-19 pandemic. Documented interventions included school and business closures, face mask mandates, directives on vaccine eligibility, eviction moratoria, and expanded unemployment insurance benefits. By providing continually updated information, *CUSP* was designed to inform rapid-response, policy-relevant research in the context of the COVID-19 pandemic and has been widely used to investigate the impact of state policies on population health and health equity. This paper introduces the *CUSP* database and highlights how it is already informing the COVID-19 pandemic response in the US.

**Conclusion:**

*CUSP* is the most comprehensive publicly available policy database of health, social, and economic policies in response to the COVID-19 pandemic in the US. *CUSP* documents widespread variation in state policy decisions and implementation dates across the US and serves as a freely available and valuable resource to policymakers and researchers.

**Supplementary Information:**

The online version contains supplementary material available at 10.1186/s12889-022-13487-0.

## Background

Coronavirus disease 2019 (COVID-19) brought more than 84 million cases and over one million deaths to the United States (US) by May 31, 2022, numbers that continue to rise [1]. Unemployment in April 2020 reached levels not seen since the Great Depression [2]. Both COVID-19 deaths and unemployment have been characterized by marked inequities in race and income [3]. At the start of the pandemic, many states adopted mitigation strategies that included stay-at-home orders and school and business closures to limit in-person contact and prevent disease transmission. In addition, states instituted face mask requirements, expanded social safety net programs such as unemployment insurance and nutrition assistance programs, and froze the eviction process to control the spread of the virus and to address economic precarity. COVID-19 prevention policies affect how quickly the disease spreads [4, 5], mortality [6], and mental health [7]. Social safety net policies may have similarly large implications for health and mortality [8–11]. Evaluating which policies most affect population health and health equity can provide essential information for policymakers as the pandemic and its consequences continue to affect people across the country and world.

The data available to policymakers, researchers, and the public shape how policymakers respond to and evaluate policy drivers of the ongoing pandemic and economic crisis. To inform policymakers and the public and facilitate rapid research, we created the *COVID-19 US State Policy* (*CUSP*) database in March 2020.

### Construction and content

We recorded the dates when each US state implemented key new social safety net, economic, physical distancing, and COVID-19 vaccine distribution policies, combined with data on existing health and social policies and information on state characteristics. To facilitate rapid dissemination and collaboration during the early weeks of the pandemic, the database was originally available as a Google Sheet. Later, we made all data and data dictionaries publicly available via the *CUSP* website [12] and GitHub [13] with detailed notes on coding decisions designed to inform researchers as they make their own coding decisions. As policies changed over time, we documented updates to the database in a publicly available changelog and also recorded the date that team members conducted a comprehensive review of each policy area. Folders with official government documentation underlying each coding decision are publicly available via Dropbox [14]. While other COVID-19 policy databases complement this work, *CUSP* offers a unique resource by providing data on the specific dates and components of health, social, and economic policy changes and in providing complete source documentation. The *CUSP* data expand upon or may be linked with other publicly available databases on COVID-19 cases and deaths [15], state health systems capacity [16], physical distancing policies [17], vaccine prioritization [18], and specific social policies like paid sick leave [19].

The team that created *CUSP* included dozens of graduate and law student volunteers working alongside research fellows and faculty members. To create *CUSP*, the research team compiled and reviewed executive orders, legislation, court orders, and directives from state government websites and reviewed media coverage to search for additional government orders. States were considered to have implemented a policy only if state governors or other state government officials or courts issued directives or executive orders or passed legislation; recommendations or guidance were not considered policy changes. The team then compared findings to any similar state tracking efforts, available through media, non-profit organizations, or other research groups, to validate policy changes, if possible. The eviction moratoria and protective measures were also validated directly by the initiating state actors, including the state supreme court and governors’ offices. A senior team member re-reviewed each date and states with no policy decisions and posted to the publicly available database with comments on the team’s coding decisions. *CUSP* publicizes the database’s revision history and invites comments through a comment portal on the *CUSP* website, which has been reviewed weekly by the study team. As pandemic response policies change in the future, we will continue to update the database, to document these changes, and to record the date of our latest comprehensive review of each policy area. However, we anticipate fewer policy changes during the months and years ahead in comparison to the rapidly evolving policies that were implemented during the initial COVID-19 crisis.

We created the *CUSP* database as a public good to both memorialize this period of political creativity and to facilitate the rapid emergency response to the COVID-19 pandemic by policymakers and researchers. Researchers may want to carefully consider the policy coding that is the best fit for their specific research question. For instance, our research team only recorded directives and orders, based on our observations that recommendations and guidance have limited efficacy, but this decision may not be suitable for every research project. We provide the *CUSP* source documentation as an additional publicly available resource to facilitate such decisions. We are prepared to keep the *CUSP* database, including its data dictionary, changelog, and source documentation, online indefinitely so that researchers and policymakers may continue to learn from COVID-19 policy decisions to inform future pandemic responses.

### Utility and discussion

#### COVID-19 prevention policies

**Fig. 1:**
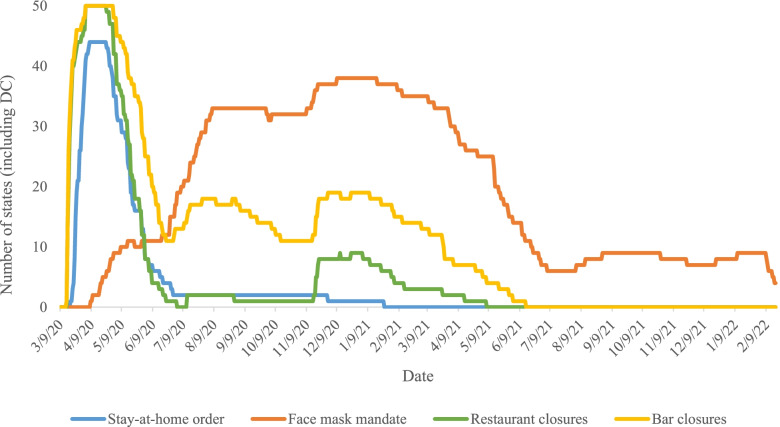
Number of states that implemented policies to prevent the spread of COVID-19 (March 9, 2020 – February 18, 2022)

*CUSP* documents the widespread variation in policy decisions and dates of implementation across the US (Supplementary Table 1). Policies designed to mitigate COVID-19 transmission such as physical distancing policies and business closure policies significantly decelerated viral spread throughout the US [4]. Our database shows that all 50 US states and the District of Columbia (DC) implemented at least one physical distancing policy [12]. For instance, 49 states and DC initiated specific business closures such as restricting restaurants to take-out and closing bars, gyms, and movie theaters (Fig. 1).

COVID-19 prevention policies were implemented in the context of longstanding inequities in health and economic outcomes by race/ethnicity, immigration status, and income. Structural racism shaped policies both before and during the pandemic that manifested in racial and economic disparities in COVID-19 exposure and mortality [20]. Using *CUSP *data on the dates that US states implemented physical distancing orders, research found that state physical distancing policies did not sufficiently mitigate inequities in barriers to physical distancing in lower-income neighborhoods [21]. Low-income workers, who are disproportionately Black and Latinx, were less likely to be able to work from home and were less protected by physical distancing policies. Similarly, research using *CUSP* housing data, developed in collaboration with Emily Benfer et al., showed that people were only able to adhere to stay-at-home orders when policies allow them to keep their homes [22]. Our database documents the dates that forty-three US states and DC instituted and lifted eviction moratoria. These data were analyzed to demonstrate the associations between eviction moratoria and COVID-19 incidence and mortality [23], mental health outcomes [24], and eviction filing rates [25].

In public indoor settings where physical distancing was not always feasible, face mask policies also reduced COVID-19 case and death growth rates [26]. *CUSP* documents when, where, and how states implemented face mask mandates from the start of the pandemic to present day. Forty-one states mandated face masks in public spaces at some point during the first year of the pandemic, yet only 8 states had mask policies in place past June 2021 (Fig. 1), following a change in guidance from the Centers for Disease Control and Prevention (CDC). Although children were not yet eligible for COVID-19 vaccination at the start of the 2021 school year [27], just 16 states mandated face masks in schools during this time, while 9 states banned face mask mandates in schools [12]. Although judicial decisions later blocked nearly all of these states from enforcing bans on face mask mandates in schools, the impact on children and teachers’ health during this period has yet to be quantified.

Research using *CUSP *data found that state-level mask orders were associated with decreased COVID-19 growth rates and demonstrated an association between greater mask adherence and reduced rates of COVID-19 transmission [28, 29]. Further investigation remains to assess the effectiveness of mask policies in the context of widespread vaccine eligibility and against the delta and omicron variants, and to evaluate how mask policies shape COVID-19 transmission during surges. These data may be particularly useful in tandem with epidemiologic data to ​​inform the distribution of high-quality masks nationwide and to establish workplace standards in high-risk settings.

Equitable COVID-19 vaccine delivery is another effective public health strategy for COVID-19 prevention. In December 2020, when the Food and Drug Administration (FDA) issued emergency use authorization for COVID-19 vaccines, CDC’s Advisory Committee on Immunization Practices (ACIP) recommended initial prioritization of health care personnel and long-term care facility residents subsequently followed by adults aged 75 and over and frontline essential workers [30]. *CUSP* documents the policy decisions states made to distribute the initial supply of COVID-19 vaccines, including the dates and phases in which certain populations became eligible for vaccination based on age, occupation, housing status, or race and ethnicity.

**Fig. 2:**
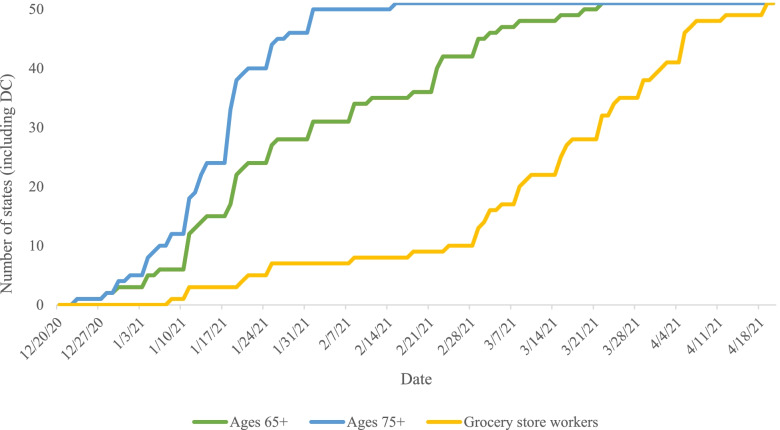
Number of states that made high-risk groups eligible for COVID-19 vaccination (December 20, 2020 – April 20, 2021). The Centers for Disease Control and Prevention’s (CDC) Advisory Committee on Immunization Practices (ACIP) recommended that COVID-19 vaccines be allocated to frontline essential workers, such as grocery store workers, prior to persons aged 65-74 years and simultaneously with persons aged 75 + years. No states followed this policy recommendation

Many states did not prioritize vulnerable populations, such as those in carceral facilities, for COVID-19 vaccination – nor did ACIP. Yet state-level vaccine prioritization of people who are incarcerated was associated with increased COVID-19 vaccination rates among this population [31]. Further, a call by federal officials from the US Department of Health and Human Services in January 2021 to adhere to age-based vaccine prioritization schemes led a majority of states to revise their policies and go against ACIP’s guidance to include essential workers in the second phase. *CUSP* data shows that 39 states prioritized adults ages 65 and older ahead of essential workers, and the remaining states prioritized these two high-risk groups simultaneously (Fig. 2). Just 3 states prioritized vaccine distribution by race and ethnicity [12]. These policy choices to deprioritize essential workers, who face high rates of exposure to COVID-19 and are more likely to be Black and Latinx, may have exacerbated existing ethnic and racial disparities in COVID-19 case and mortality rates [32]. Other structural barriers including access to a telephone or Internet to schedule vaccine appointments and paid time off [33] to recover from vaccine side effects shape access to vaccination and drive racial and income inequities. Loss of wages from lack of paid time off can drive risks such as food and housing insecurity [21].

#### Policies to reduce economic precarity

**Fig. 3:**
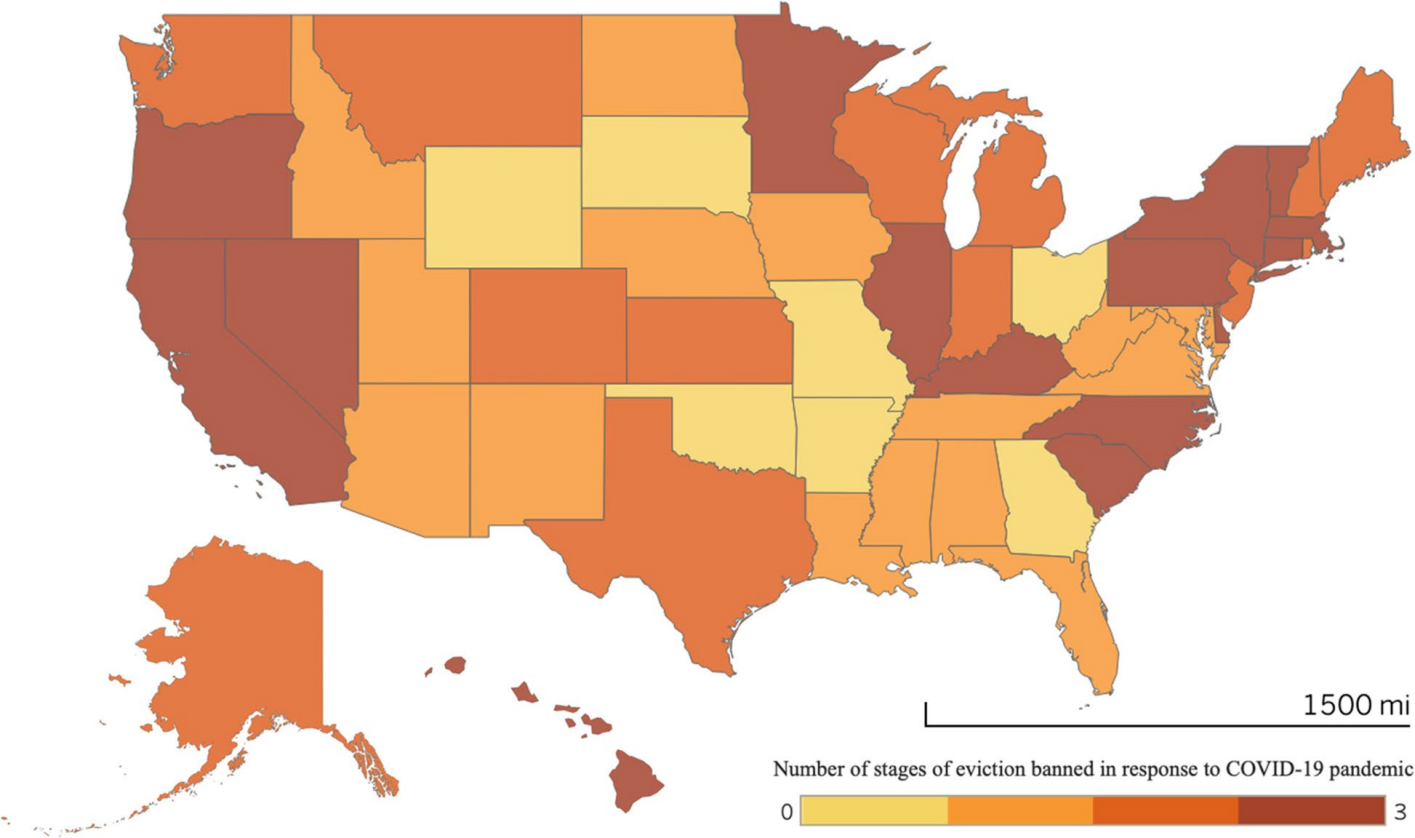
Stages of eviction banned by US states in response to the COVID-19 pandemic. AK and HI not shown to scale. CUSP tracks the dates that states banned certain stages of the eviction process: 1) initiation (i.e. notice or filing) of eviction; 2) eviction hearings; 3) enforcement of orders of eviction. This map depicts US states that banned some combination of these stages in response to the COVID-19 pandemic, ranging from none (lightest shade) to all (darkest shade)

Social safety net policies to support financial stability and housing and food security are equally as important as COVID-19 prevention policies in shaping the short- and long-term health and economic consequences of the COVID-19 pandemic. For instance, homelessness is associated with worse physical and mental health [11, 34]. *CUSP *documents housing policy data from the COVID-19 Eviction Moratoria & Housing Policy database that tracks the dates of eviction bans in each state for each stage of the evictionprocess [22]. For the first time in US history, 26 states and DC banned notice and/or filing of evictions, and 30 states and DC banned eviction hearings and/or enforcement of evictions (Fig. 3). Additionally, 13 states and DC banned the collection of late fees for delayed rent payments [12].

Food insecurity is similarly associated with worse physical and mental health [10]. *CUSP *documents state-level policies in response to COVID-19 that aimed to address food insecurity in the context of the pandemic. To expand access to the Supplemental Nutrition Assistance Program (SNAP), all 50 states and DC used waivers to allow emergency SNAP allotments to existing SNAP households, and 50 states and DC used Pandemic Electronic Benefits Transfer (EBT) to provide SNAP assistance to households with children who would normally receive free or reduced-price meals through school [12].

**Fig. 4:**
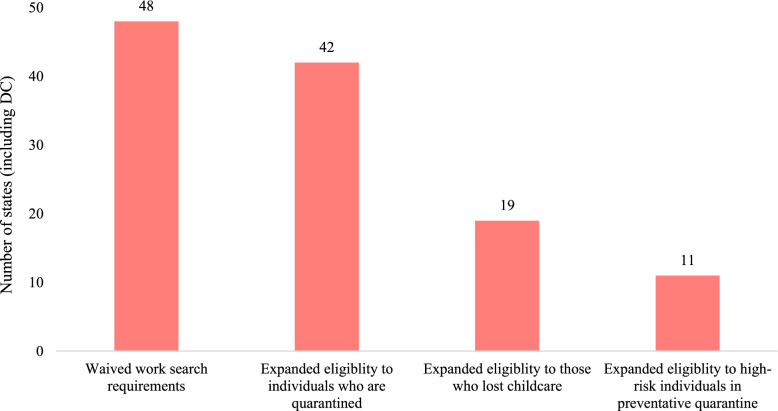
States that expanded unemployment insurance policies in response to the COVID-19 pandemic

The provision of unemployment insurance (UI) benefits is also likely to play an important role in economic stability and mental health [35, 36]. All 50 states and DC made at least one policy change to expand access to UI; 48 of these jurisdictions waived work search requirements, 44 waived the waiting period prior to UI initiation, 42 expanded UI eligibility to individuals in isolation with COVID-19 or caring for someone with COVID-19, 19 expanded UI eligibility to individuals who lost child care, and 11 expanded UI eligibility to high-risk individuals in preventative quarantine (Fig. 4). Federal policies via the Coronavirus Aid, Relief, and Economic Security (CARES) Act also expanded UI and authorized a $600 weekly supplement to state UI benefits.

*CUSP *data that document policies to reduce economic precarity in the context of the COVID-19 pandemic have critical implications for health and health equity. Although US Census Household Pulse Survey data revealed that low-wage workers in the US were most likely to report missing work due to COVID-19, these workers were least likely to have access to paid leave [37]. As a result, many low-income workers who missed work due to COVID-19 were forced to forgo wages and thus were sometimes unable to afford enough food to eat. Similarly, a pre-print using *CUSP* data found that living in a state with at least a $12 minimum wage was associated with reduced food insecurity, as was having access to paid leave [38]. However, research found that UI receipt between April 1 and November 11, 2020 was associated with a 35% reduction in food insecurity, and the CARES Act $600 per week supplement to UI was associated with a larger reduction in food insecurity [35]. Policies such as UI, paid leave, and increased federal minimum wage may help alleviate food insecurity and improve health.

Further areas of investigation using *CUSP* data may involve evaluating the variation in UI dollar amounts and duration by state and assessing the health impact of terminating expanded UI benefits to inform long-term UI reform. There are also gaps in the literature describing the relationship between economic policies and COVID-19 as new, more transmissible variants of COVID-19 have spread across the US.

## Discussion

*CUSP* was established as a comprehensive, publicly-available policy database of US state health, social, and economic policies implemented in response to the COVID-19 pandemic. *CUSP *extends upon the work of existing policy databases, such as the Oxford COVID-19 Government Response Tracker, by tracking a wider range of state-level policy measures [39]. Other sources, including the Council of State Governments [40], have compiled state executive orders in response to the COVID-19 pandemic, but have not interpreted these documents or extracted dates of implementation.

Future research using *CUSP* data may explore how state vaccine prioritization and other COVID-19 prevention policies shaped inequities in health outcomes. Additionally, research using *CUSP* economic policies may evaluate the public health impact of expanding unemployment insurance and paid leave, especially during periods of economic precarity. Following the 2008 financial crisis, a decade of research emerged using data from this time period, intended to inform future economic crises. We anticipate that *CUSP* may similarly facilitate long-term learning about how policies in response to COVID-19 shaped a range of health and economic outcomes in order to guide future pandemic response. For instance, COVID-19-related policies may have contributed to record-breaking increases in firearm homicide [41] and drug overdose [42] during the pandemic and these potential relationships warrant further study.

*CUSP* data have several limitations. Although our team consistently reviews changing policies and updates *CUSP* on a frequent basis, it is possible that certain policies evolved more recently. To be transparent about our data collection process amidst a rapidly changing policy environment, we note the date we last conducted a comprehensive review of each policy area in *CUSP*, and we document state-by-state updates in our changelog. Additionally, data extraction from executive orders and government websites – many of which are not text searchable – was done by hand by members of our research team; as such, these data may contain human errors. However, we minimized the likelihood of error through an extensive data review process within our team and in comparison to other policy resources. We recommend that researchers or policymakers triangulate the *CUSP* data with complementary data from other sources as well. An additional limitation to the *CUSP* database is that it does not capture the effectiveness of policy implementation. However, *CUSP* data may be linked to other data sources and used in policy analyses, as it has been in nearly two hundred studies as of May 2022, to evaluate the effects of policies on health equity and population health in the context of the COVID-19 pandemic.

## Conclusion

There has been a proliferation of state policy responses to the joint infectious disease, economic, and mental health crises facing people across the US during the COVID-19 pandemic. At a time of widespread population health and financial vulnerability, state policy decisions are likely to have consequences that extend well beyond COVID-19 transmission, including for mental health and interpersonal violence, as well as for financial insecurity, homelessness, food insecurity, and access to health care. *CUSP* data can help researchers and policymakers document the widespread and continued impact of these state policies.

## Supplementary Information

**Additional file 1: Supplementary Table 1.** Key US pandemic response policies for COVID-19 prevention and to reduce economic precarity.

## Data Availability

The dataset supporting the conclusions of this article is available in the COVID-19 US State Policy Database repository, https://github.com/USCOVIDpolicy/COVID-19-US-State-Policy-Database [13].

## Abbreviations

ACIP: Advisory Committee on Immunization Practices
CDC: Centers for Disease Control and Prevention
CARES: Coronavirus Aid, Relief, and Economic Security
COVID-19: Coronavirus disease 2019
CUSP: COVID-19 US State Policy
DC: District of Columbia
EBT: Electronic Benefits Transfer
FDA: Food and Drug Administration
UI: Unemployment insurance
US: United States
