# Supplementary material for: A database of US state policies to mitigate COVID-19 and its economic consequences
Source: BMC Public Health. 2022 Jun 4;22:1124. doi: 10.1186/s12889-022-13487-0 (PMC9166228; doi:10.1186/s12889-022-13487-0)
Supplement: Supplementary file 1 — Additional file 1: Supplementary Table 1. Key US pandemic response policies for COVID-19 prevention and to reduce economic precarity. [file 12889_2022_13487_MOESM1_ESM.docx]

**Supplementary Table 1**. Key US pandemic response policies for COVID-19 prevention and to reduce economic precarity

| **Policy Area** | **Policies** |
| --- | --- |
| State of Emergency | State of emergency start/end dates |
| Stay at Home | Stay-at-home order start/end dates |
| Closures | Restaurant closure start/end dates |
|  | Bar closure start/end dates |
|  | Casino closure start/end dates |
|  | Day care closure start/end dates |
|  | School closure start dates |
|  | Non-essential business start/end dates |
| Face Masks | Mask mandate start/end dates |
|  | Mask mandate exemptions |
|  | Mask mandate in schools |
|  | Mask mandate bans |
| Interstate Travel Quarantine | Quarantine requirement for interstate travelers start/end date |
| Housing | Eviction moratoria start/end date |
|  | Eviction initiation ban start/end date |
|  | Eviction hearing ban start/end date |
|  | Eviction enforcement ban start/end date |
|  | Limitations for COVID-19 hardship and non-payment |
|  | CARES Act pleading start/end date |
|  | CDC moratorium start/end date |
|  | Late fee ban start/end date |
| Utilities | Utilities shutoff moratoria start/end date |
| Unemployment Insurance | Date waiting period waived |
|  | Date work search requirement waived/reinstated |
|  | Eligibility expansions |
|  | Weekly maximum amounts |
|  | Maximum durations |
|  | Extended Benefits program start/end date |
|  | Date federal unemployment benefit programs ended |
| Workplace Protections | OSHA-approved state plans |
|  | Aerosol transmissible disease standards |
|  | Air or ventilation standards |
|  | Paid leave expansions |
|  | Workers’ compensation expansions |
|  | Liability protections |
|  | Data reporting |
| Minimum Wage | Minimum wage amounts 2019-2020 |
| Food Security | SNAP waivers |
| Healthcare Delivery | Medicaid telehealth coverage expansions |
|  | CHIP premium non-payment lockout suspensions |
|  | Elective medical procedure suspension start/end date |
|  | Abortion access restrictions |
| Racial Disparities | Data reporting by race/ethnicity |
| Incarceration | State prison visitation ban start/end date |
|  | Date copays waived for incarcerated persons |
